# HLA-DR in Cytotoxic T Lymphocytes Predicts Breast Cancer Patients' Response to Neoadjuvant Chemotherapy

**DOI:** 10.3389/fimmu.2018.02605

**Published:** 2018-11-13

**Authors:** Diana P. Saraiva, António Jacinto, Paula Borralho, Sofia Braga, M. Guadalupe Cabral

**Affiliations:** ^1^CEDOC, NOVA Medical School, Faculdade de Ciências Médicas da Universidade Nova de Lisboa, Lisbon, Portugal; ^2^Instituto CUF de Oncologia, Lisbon, Portugal; ^3^Faculdade de Medicina da Universidade de Lisboa, Lisbon, Portugal; ^4^Department of Biomedical Sciences and Medicine, Universidade do Algarve, Faro, Portugal

**Keywords:** breast cancer, cytotoxic T lymphocytes, HLA-DR, prediction, neoadjuvant chemotherapy

## Abstract

Prediction of breast cancer response to Neoadjuvant Chemotherapy (NACT) is an urgent need to promptly direct non-responder patients to alternative therapies. Infiltrating T lymphocytes, namely cytotoxic T lymphocytes (CTLs) have been appointed as predictors of response. However, cancer cells have the ability to dampen CTLs' activity and thus, the prognostic value of the CTLs, *per se*, is debatable. Here, we disclose that more than the occurrence of CTLs, it is their activation state, revealed by HLA-DR expression, that can accurately predict response to NACT. Flow cytometry analysis of breast cancer biopsies showed that the frequency of CTLs and other lymphocytes were similar regardless disease stage and between NACT responders and non-responders. However, only breast cancer patients without axillary lymph node metastasis and NACT responders have HLA-DR^hi^ CTLs. Interestingly, HLA-DR levels in tumor CTLs is correlated with HLA-DR levels in systemic CTLs. These HLA-DR+ CTLs produce IFN-γ and Granzyme B, enlightening their effector and probable anti-tumor activity profile. Moreover, the level of HLA-DR in CTLs is negatively correlated with the level of HLA-DR in T regulatory lymphocytes and with immunosuppressive and pro-tumor molecules in the tumor microenvironment. Hence, HLA-DR levels in CTLs is a highly sensitive and specific potential predictive factor of NACT-response, which can be assessed in blood to guide therapeutic decisions.

## Introduction

Breast cancer remains one of the main causes of cancer-related deaths in women worldwide (1). The disease can be divided in three different subtypes—hormone-positive (estrogen and/or progesterone—ER+ and/or PR+), human epidermal growth factor receptor 2 (HER2) positive and triple negative breast cancer (TNBC), which lacks the three mentioned markers. In the past years, advances in breast cancer treatment have been made, namely with the introduction of preoperative neoadjuvant chemotherapy (NACT) in selected cases of advanced tumors (with tumor size larger than 2 cm and/or disease extension to axillary lymph node) or inflammatory breast cancer. This treatment is effective in reducing the size of the primary tumor, allowing breast conservation in selected cases, and some patients achieve a pathological complete response (pCR) (2). However, this only happens in less than 50% of the patients (3, 4) and residual disease after NACT is a strong predictor of relapse (2, 5). Hence, it is essential to find a good marker of response to NACT, in order to promptly direct patients to alternative therapies, therefore avoiding the misuse of resources and the potential toxicity associated with NACT.

Increasing evidence suggests that anti-cancer chemotherapy is influenced by the immune system (6). Indeed, tumors can be heavily infiltrated by immune cells, in particular, tumor infiltrating lymphocytes (TILs), which have been associated with good prognosis in various cancers, including breast cancer (6). Several reports have been advocating that TILs, especially cytotoxic T lymphocytes (CTLs), due to their anti-tumor cytotoxic activity, could serve as a robust marker for predicting pCR rate after NACT (7). However, even with an effort to standardize TILs evaluation (8), data are still conflicting, and other reports describe that TILs and their subsets could not show any predictive value, particularly in hormone-positive subtype (6), possibly because these patients are further treated with endocrine therapy for years. Furthermore, T regulatory cells (Tregs), by suppressing the effector immune response, are normally associated with poor prognosis. However, they are still described to have an ambiguous role (9).

Actually, tumor cells have mechanisms to escape the immune system, and the activity of CTLs can be hampered by them. For instance, tumors may express inhibitory molecules like programmed death-ligand 1 (PD-L1) which bind the inhibitory receptor programmed death 1 (PD-1) in CTLs, inducing negative regulatory pathways that limit the activity of these cells (10, 11). Other inhibitory immune checkpoints, such as cytotoxic T-lymphocyte-associated protein 4 (CTLA-4) and the secretion of immunosuppressing molecules, such as IL-10, TGF-β or Indoleamine 2, 3-Dioxygenase (IDO), could also directly or indirectly (by recruiting and activating Tregs), negatively impact CTLs' activity (10, 11).

Considering the aforementioned, the presence of CTLs *per se* provides limited information regarding NACT outcome, and the activation level of these cells, as a result of the overall tumor immune status, should be taken into account in order to better predict response to NACT.

HLA-DR is recognized as a marker of T cell activation (12, 13) and has been shown to be increased in CTLs in autoimmune diseases (14) and in patients with HIV infection (15).

In this work, by flow cytometry analysis of fresh samples, we demonstrated that a subset of CTLs expressing HLA-DR is enriched in breast cancer without axillary lymph node metastasis comparing with breast cancer with axillary lymph node metastasis, although the average frequency of CTLs is similar between groups. Furthermore, biopsies from NACT responders also have HLA-DR^hi^ CTLs, while no differences were observed concerning the density of CTLs between NACT responders and non-responders. Interestingly, the profile of HLA-DR^hi^ CTLs was negatively correlated with high levels of pro-tumor and immunosuppressive molecules in the tumor immune microenvironment.

Therefore, we propose that HLA-DR+ CTLs have the potential to be used in clinical settings to predict breast cancer patients' response to NACT, with the advantage of being easily accessed in blood.

## Materials and methods

### Patient samples

Forty-eight fresh biopsies and ninety-six non-matched surgical samples of breast cancer (BC) patients were collected in saline solution. Matched peripheral blood from 31 BC patients was collected in Vaccutainer tubes with EDTA (BD Biosciences). Whole blood from 13 healthy donors was also collected for comparison studies. For *in vitro* studies, patients' peripheral blood mononuclear cells (PBMCs) were isolated by a Ficoll gradient (Merck Milipore) and PBMCs from buffy coats of 5 healthy donors, donated by Instituto Português do Sangue e da Transplantação, were used for control purposes. Formalin-fixed paraffin embedded (FFPE) tissue was collected from 12 matched patients divided according to the axillary lymph node invasion status and response to neoadjuvant chemotherapy (NACT). A summary of the patients characteristics are given in Table 1.

**Table 1:** Characteristics of patients enrolled in this study (age, body mass index, and menopause).

Age	Median−58 (range: 35–87)
Body mass index (BMI)	Median−26.03 (range: 17.04–39.74)
Post-menopause	62.30%
ER+ (PR –/+)	68%
HER2+ including triple positive breast cancer	16%
TNBC	16%
Grade	G1–8.26%
	G2–58.68%
	G3–33.06%
Ki67	Median−21.75% (range: 2–98.4%)
Dimension (mm)	Median−25 (range: 7–90)
Axillary lymph node invasion status	Negative−51.09%
	Positive−48.91%
Response (if NACT)	Good response−43.33%
	Bad response−56.67%

*Clinical data, such as subtype of breast cancer, grade, Ki67, tumor dimension, node status and response to treatment are also described*.

Fresh samples were handled within 1-day post collection. Fresh tumors and biopsies were mechanically dissociated with a BD Medicon (BD Bioscience), filtered and washed once with PBS 1X. PBMCs after isolation were cryopreserved in 90% fetal bovine serum (FBS, Biowest) and 10% dimethyl sulfoxide (DMSO, Sigma Aldrich) until further use.

Samples were gathered from Hospital CUF Descobertas, Hospital Prof. Doutor Fernando Fonseca and Hospital de Vila Franca de Xira. For each patient, written informed consent and approval by the Ethical Committee of the hospitals and of the NOVA Medical School were obtained. The study is in compliance with the Declaration of Helsinki.

### Antibodies

For flow cytometry analysis, tumor and blood samples were stained with a cocktail of monoclonal mouse anti-human conjugated antibodies (mAbs): anti-CD45-PercP (clone HI30), anti-CD3-PercP (HIT3a), anti-CD3-APC (UCHT1), anti-CD19-PE (HIB19), anti-CD15-PE (HI98), anti-CD161-FITC (HP-3G10), anti-CD4-FITC (OKT4), anti-CD8-PE (HIT8a), anti-HLA-DR-APC (L243), anti-CD127-PE-Cy7 (A019D5), anti-CD1c-APC-Cy7 (L161), anti-CD163-PE (GHI/61), anti-CD206-APC-Cy7 (15–2), anti-PD-1-FITC (EH12.2H7), anti-PD-L1-APC (29E2A3), anti-CTLA4-PE (L3D10), anti-CD69-APC-Cy7 (FN50), anti-Tim3-APC (F38-2E2), anti-IL-8-APC (E8N1), anti-IFN-γ-PE (4S.B3), anti-IFN-γ-APC-Cy7 (4S.B3), anti-Granzyme B-FITC (QA16A02), anti-IL-1β-FITC (JK1B-1), anti-IL-2-PE-Cy7 (MQ1-17H12), anti-IL-6-APC (MQ2-13A5), anti-IL-17-FITC (BL168), anti-IL-23/IL-12-PE (C11.5), anti-TGF-β-APC (TW4-6H10), all from Biolegend; anti-IDO-PE (eyedio) and anti-IL-10-FITC (BT-10), both from eBioscience; anti-CD25-PE (MEM-181) and anti-CD11b-FITC (LT11) from ImmunoTools.

The antibodies used for immunofluorescence were: mouse monoclonal anti-human CD8 (32-M4) from Santa Cruz Biotechnology and rabbit polyclonal anti-human HLA-DRA from Sigma Aldrich.

### Flow cytometry

BC samples were stained with BD Horizon™ Fixable Viability Stain 450 (BD Biosciences) and with the cocktail of antibodies, fixed and permeabilized with Fix/Perm kit (eBiosciences) followed by intracellular staining. For whole blood, staining with antibodies was followed by a step of red blood cells lysis with RBC lysis buffer (Biolegend).

For immunophenotyping we classified cytotoxic T lymphocytes as CD45+/CD3+/CD8+; helper T lymphocytes as CD45+/CD3+/CD4+; regulatory T lymphocytes as CD45+/CD3+/CD4+/CD25^hi^/CD127^lo^; B lymphocytes as CD45+/CD19+; NK cells as CD45+/CD161+; M2 macrophages as CD45+/CD11b+/CD163+/CD206+; M1 macrophages as CD45+/CD11b+/CD163negative/CD206negative; dendritic cells as CD45+/CD1c+; and neutrophils as CD45+/CD15+.

Data was acquired in BD FACS Canto II cell analyzer with FACSDiva Software v8.0.1 (BD Biosciences) and the results were analyzed using FlowJo software v10. The data is presented as percentage of the populations in respect to the gate of single cells, following the gate strategy represented in Figure S1. To analyze the expression levels of HLA-DR in CTLs or Tregs, we considered the median fluorescent intensity of positive population and normalized it to the negative population, as previously described (16). The negative population was superimposed with the unstained control.

### *Ex vivo* stimulation assay

Isolated PBMCs were cultured in 96 well-plates with U bottom (Sigma Aldrich) with RMPI-1640 (Gibco) supplemented with 10% FBS and 1% Penicillin/Streptomycin (GE Healthcare). Stimulation was performed during 4 h (or overnight for HLA-DR expression) with 35 ng/mL of phorbol 12-myristate 13-acetate (PMA, Sigma Aldrich) and 1 μg/mL of ionomycin (Merck Milipore). Brefeldin A (Biolegend) was added for 4 h to stop the extracellular transport. Cells were collected and stained with mAbs and analyzed by flow cytometry.

### Immunofluorescence

Immunofluorescence was performed in formalin-fixed paraffin embedded tissues (FFPE). Paraffin was removed in xylene (10 min) and passed through a gradient of alcohol (100, 96, and 70%) and dH_2_O. Antigen retrieval was performed in 2 cycles of 15 mins each with 1 mM EDTA (Sigma Aldrich) with 0.05% Tween-20 (Sigma Aldrich) in the microwave (900W). The slides were permeabilized with PBS 1X + 0.3% Triton X-100 (ACROS Organics) and blocked with PBS 1X + 0.1% Triton X-100 + 1% bovine serum albumin (BSA, Sigma Aldrich) + 1.5% goat serum (Sigma Aldrich). Staining with primary antibody (1:100) was performed overnight at 4°C followed by the incubation with secondary antibody for 2 h in the dark at room temperature. Counterstaining was performed with 4′,6- diamidino-2-phenylindole (DAPI; 0.001 mg/mL in PBS, Sigma-Aldrich) and the slides were mounted in Fluorescent Mounting Media (DAKO). Images were taken in a confocal microscope (LSM710, Zeiss) and analyzed in Fiji software (17).

### ELISA

The patients' plasma was isolated from whole blood and frozen for further cytokine analysis. The quantity of secreted cytokines was measured using ELISA technique, namely human IL-10 (ImmunoTools) and IFN-γ (Biolegend) kits were used according to the manufacturer's instructions. Cytokine concentration was calculated using the specific standard curves.

### Cell sorting

PBMCs were stained with the viability dye followed by the mAbs anti-CD3-PercP, anti-CD8-PE and anti-HLA-DR-APC. Cells were sorted in CD3+/CD8+/HLA-DR+ and CD3+/CD8+/HLA-DRnegative in a FACS Aria III (BD Biosciences) with an efficiency of 98% (Figure S2).

### qRT-PCR

Total RNA of sorted cells was extracted using RNeasy Micro Kit (Qiagen) and reverse transcribed with Transcriptor High Fidelity cDNA synthesis kit (Roche). Quantitative real-time PCR (qRT-PCR) was performed with several primers, described in Table 2, using Roche LightCycler 480 and FastStart Essential DNA Green Master Mix (Roche). Cyclic conditions were: 95°C for 10 min, followed by 45 amplification cycles, each consisting of 10 s at 95°C, 10 s at 56°C, and 20 s at 72°C, and finally a melting step of 10 s at 95°C, 60 s at 65°C, and 1 s at 97°C.

**Table 2:** Genes assessed with qRT-PCR and the primers used.

**Gene**	**Forward primer**	**Reverse primer**
*TNFα*	AGATGATCTGACTGCCTGGG	CTGCTGCACTTTGGAGTGAT
*Granzyme B*	GGGGGACCCAGAGATTAAAA	CCATTGTTTGGTCCATAGGAG
*Perforin*	GCAATGTGCATGTGTCTGTG	GGGAGTGTGTACCACATGGA
*Eomes*	CAGCACCACCTCTACGAACA	CGCCACCAAACTGAGATGAT
*RPL13A*	TGCGTCTGAAGCCTACAAGA	TCCGTAGCCTCATGAGCTGTT

The relative mRNA levels were normalized against the values obtained for the housekeeping gene RPL13A and calculated by the formula 2^−ΔCt^ × 1000 that gives us the number of mRNA molecules of the gene of interest per 1000 molecules of the endogenous control (17, 18).

### *In vitro* co-culture assay

HS 578T cell line was maintained for 4 days in DMEM (Biowest) with 10% FBS and 1% Penicillin/Streptomycin. After that period, the supernatant and cells were harvested for the co-culture. The supernatant was further centrifuged at 2000 rpm for 5 min to eliminate cellular debris and possible cellular antigens. PBMCs were plated on a 96 well plate U bottom in 4 conditions: monoculture, with the addition of the supernatant from HS 578T culture, with a canonical stimulus of PMA and ionomycin, and in co-culture on a ratio of 20:1 (PBMCs:HS 578T). PBMCs were collected 48 h after and analyzed by flow cytometry.

### Statistical analysis

Statistical analysis was performed in GraphPad Prism v6 and statistical significance was considered for *p* < 0.05. Comparison between samples was performed by a nonparametric Mann-Whitney test and correlations were calculated with Spearman *r*-test. ROC curves were performed to assign a threshold to divide NACT responders from non-responders. This analysis was executed for both HLA-DR expressing CTLs and Tregs. The determined area under the curve, sensitivity and specificity were taken into account. The cut-off point for the HLA-DR expression level in CTLs was a parameter also determined by ROC curve analysis, considering the expression value that corresponded to the maximum of sensitivity and specificity. Paired *t*-test was used for the analysis of the co-culture assay.

## Results

### Clinical and pathological characteristics

For this study, 144 fresh samples (137 pre-treatment - 48 biopsies and 89 surgical specimens - and 7 post-NACT surgical specimens) were collected prospectively. Data from these patients are displayed in Table 1. Most patients have estrogen receptor (ER) positive breast cancer, have high body mass index and are post-menopausal.

These samples, except the post-NACT specimens, were used in the first phase of the study for a global analysis of tumor infiltrating immune populations. Then, aiming to find an adequate biomarker of response to NACT, from these samples, we used the first consecutive biopsies (*n* = 30) of patients that were selected for NACT and related the activation status of their lymphocytes with their response to treatment.

### HLA-DR-expressing T lymphocyte populations can distinguish breast cancer axillary lymph node invasion status

**Figure 1:**
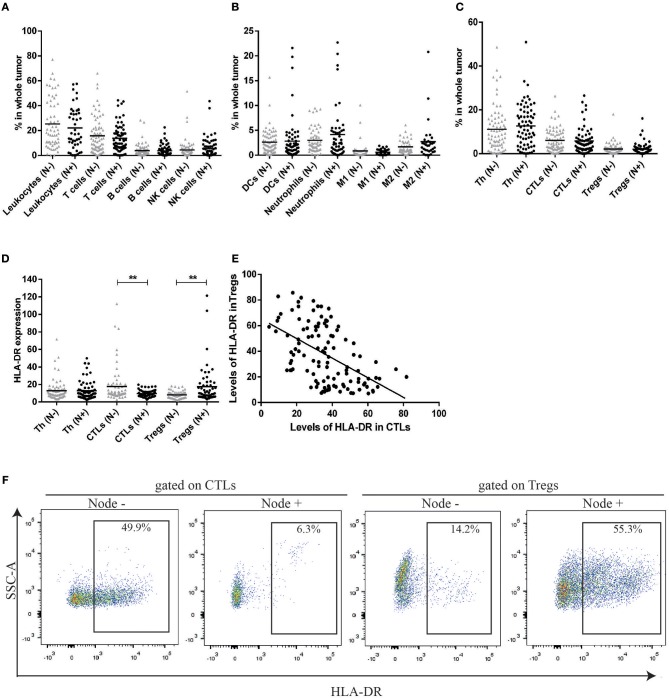
HLA-DR expression in CTLs and Tregs can distinguish between patients without axillary lymph node metastasis (N–) and patients with axillary lymph node metastasis (N+) contrarily to the percentage of distinct cell populations. **(A)** Percentage of leukocytes, T cells, B cells and NK cells in the tumor bulk from patients without axillary lymph node metastasis (N–, gray triangles, *n* = 70, mean) and patients with axillary lymph node metastasis (N+, black dots, *n* = 67, mean). **(B)** Percentage of dendritic cells (DCs), Neutrophils, M1 and M2 macrophages in the same populations. **(C)** Percentage of distinct T cell populations (helper T cells - Th, cytotoxic T cells - CTLs and regulatory T cells - Tregs) in the tumor from both groups of patients. The percentage of each population was obtained in respect to the gate in single cells (Figure S1). **(D)** Level of HLA-DR in Th, CTLs and Tregs in the tumor from patients with and without axillary lymph node invasion, expressed as the median fluorescent intensity of positive population normalized relatively to negative population (**p < 0.01). **(E)** Correlation between HLA-DR+ CTLs and HLA-DR+ Tregs in breast cancer samples (Spearman *r* = −0.54, *p* < 0.0001). **(F)** Representation of a flow cytometry analysis of HLA-DR expression gated on CTLs and Tregs and differences between a tumor from a patient without axillary lymph node metastasis and a tumor from a patient with axillary lymph node metastasis.

In the first phase of the study, and to get insight into the composition and the role of immune infiltrate in breast cancer (BC), we used a flow cytometry multipanel and analyzed several immune populations (gate strategy in Figure S1) in 137 BC patients prior to treatment implementation (48 biopsies and 89 surgical specimens). First, these patients were divided according to the axillary lymph node invasion status. Patients without axillary lymph node metastasis had a similar immunophenotype when compared with patients with axillary lymph node metastasis (Figures 1A–C). Indeed, although there was high heterogeneity in the degree of immune infiltration between patients, the average value of the frequency of T lymphocytes, B lymphocytes, NK cells (Figure 1A), neutrophils, dendritic cells, M1 and M2 macrophages (Figure 1B) infiltrating the tumor, were similar between the two groups of patients. Additionally, the average value of the frequency of T lymphocyte populations—helper (Th), cytotoxic (CTLs), and regulatory (Tregs)—were similar between these two groups (Figure 1C).

Then, we considered the activation state of T lymphocyte populations, by the analysis of established T cell activation markers, CD69 and HLA-DR. Interestingly, this analysis revealed significant differences between patients without axillary lymph node invasion and patients with axillary lymph node invasion, regarding the expression of HLA-DR. Namely, HLA-DR expression level was higher in CTLs (*p* = 0.008) and lower in Tregs (*p* = 0.001) in patients without axillary lymph node metastasis when compared to patients with axillary lymph node metastasis (Figures 1D,F). Furthermore, the expression of HLA-DR in CTLs was negatively correlated with the expression of this marker in Tregs (*r* = −0.54, *p* < 0.0001, Figures 1E, 6).

No differences were observed between patients of the two groups regarding HLA-DR expression in Th cells, but we did not discriminate between Th subsets (e.g., Th1, Th2, Th17), that might influence cancer differently.

The analysis of CD69 expression in T lymphocyte populations did not lead to the same result obtained by the analysis of HLA-DR (data not shown). This may be due to the fact that CD69 is an early, transiently expressed, activation marker of T cells (18), while HLA-DR is increased later in the activation process of T cells (19), remaining in the cell surface.

Besides dividing the patients regarding the presence or absence of axillary lymph node invasion, we have also divided them regarding breast cancer subtype (ER+, HER2+, or TNBC), grade (G1, G2, or G3), age (<50 years old or >50 years old), tumor dimension (<20 or >20 mm) and body mass index (low, normal or overweight). HLA-DR expression in T cell populations was not significantly different in these other comparisons (results not shown).

Thus, independently of BC subtype, expression of HLA-DR in distinct T cell populations can differentiate patients with axillary lymph node metastasis (HLA-DR^hi^ Tregs) from patients without axillary lymph node metastasis (HLA-DR^hi^ CTLs). These results suggested that more than simply the presence and/or the quantity of immune cells within the tumor immune microenvironment, the quality (i.e., the activation state) of certain immune cells, namely CTLs and Tregs, might be relevant for cancer progression.

### HLA-DR^hi^ CTLs are associated with response to neoadjuvant chemotherapy

**Figure 2:**
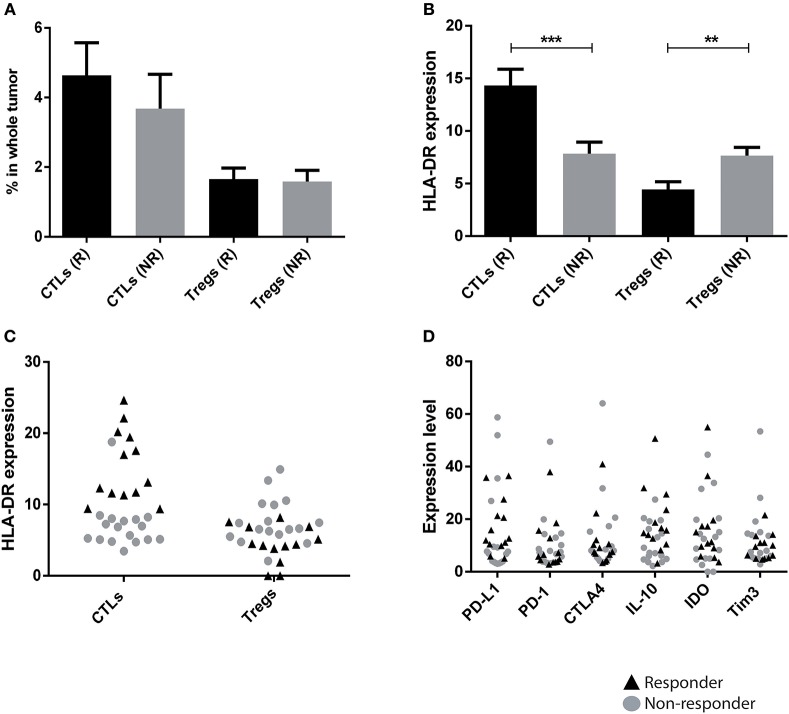
HLA-DR level in CTLs is a predictive factor of patients' response to neoadjuvant chemotherapy (NACT). **(A)** Percentage of CTLs and Tregs in biopsies from breast cancer patients that responded to NACT (R, black bar, *n* = 13, mean ± SD) and that had no response (NR, gray bar, *n* = 17, mean ± SD). The percentage of each population was obtained in respect to the gate in single cells (Figure S1). **(B)** Level of HLA-DR in CTLs and Tregs in responders and non-responders, expressed as the median fluorescent intensity of positive population normalized relatively to negative population (**p < 0.01, ***p < 0.001). **(C)** HLA-DR expression in CTLs and Tregs with data for responders and non-responders plotted in a single column (black triangles and gray dots, respectively). **(D)** The same analysis as in **(C)** was performed for other markers that theoretically could be used in the clinic: PD-L1, PD-1, CTLA4, IL-10, IDO and Tim3.

Considering that the HLA-DR expression in T lymphocyte populations allows the distinction of axillary lymph node invasion status, we asked if this trait could also be useful to predict patients' response to NACT. NACT was identical in all patients and was composed of 4 cycles of anthracyclines (doxorubicin) and cyclophosphamide, followed by 12 weeks of paclitaxel. Only the addition of trastuzumab during paclitaxel administration differed in HER2 patients. Biopsies from 30 patients that underwent NACT were here analyzed (pre-treatment) by flow cytometry and, after the treatment, patients were divided in responders and non-responders, according to their radiological and pathological outcome. NACT responders were classified as patients that achieved a pathological complete response (pCR, *n* = 6) or that had a pathological partial response with less than 10% of the initial tumor still present after treatment and without axillary lymph node involvement (*n* = 7). NACT non-responders included patients that still maintained more than 50% of the initial tumor mass after treatment (*n* = 6), or patients that developed brain, liver and/or lung metastasis during NACT (*n* = 3) or patients that had an early relapse after NACT (*n* = 8). Patients considered NACT responders were either ER+ (30.77%), HER2 (38.46%), or TNBC (30.77%). Interestingly, NACT responders showed higher levels of HLA-DR in CTLs (*p* = 0.0003) and lower levels of HLA-DR in Tregs (*p* = 0.009) when compared to the non-responders (Figure 2B). Again, the percentage of T lymphocyte populations was not statistically different between the two groups (Figure 2A). This finding is independent of BC subtype (Figure S3). Indeed, the percentage of CTLs, *per se*, was not sufficient to discriminate responders from non-responders, across all three BC subtypes, while HLA-DR expression levels in CTLs can segregate both groups, especially in ER+ and TNBC (Figure S3). In HER2 breast cancer, HLA-DR levels in CTLs was still higher in responders, comparing with non-responders, although not statistically significant. This may be explained by the fact that we only have 3 samples from non-responders, including the only sample where the HLA-DR^hi^ in CTLs did not correspond to a NACT responder.

In order to verify the predictive value of HLA-DR expression in CTLs for NACT response, in general, we plotted the levels of HLA-DR in CTLs and Tregs, of the total 30 biopsies in one single column, regardless of the response (Figure 2C). Excitingly, even with a small sample size, a robust separation of responders (black triangles) and non-responders (gray dots) was observed according to the expression level of HLA-DR, especially in CTLs (Figure 2C).

Additionally, ROC curve analysis was performed, leading to a statistically valid cut-off point for the HLA-DR expression level in CTLs (8.943 - value above which patients are NACT responders) and in Tregs (5.655 - value beneath which patients are NACT responders). For HLA-DR-expressing CTLs, the area under the ROC curve was 0.959, the sensitivity was 94.12% and the specificity was 100%. For HLA-DR-expressing Tregs, the area under the ROC curve was 0.849, the sensitivity was 81.25% and the specificity was 75%. These results highlighted that, mainly, HLA-DR-expressing CTLs evaluated in biopsies could predict response to NACT with accuracy. To clarify the few borderline cases, the analysis of HLA-DR+ Tregs can also be useful, since there is a strong negative correlation between both markers (Figure 1E).

It has been reported that chemotherapeutic agents are able to alter the immune context of breast cancer, usually boosting anti-tumor CTLs' activity (20). Thus, post-NACT surgical samples, non-matched with the pre-NACT samples, were analyzed by flow cytometry. These specimens revealed that non-responders have infiltrating CTLs with low levels of HLA-DR (*p* = 0.04) and Tregs with high levels of HLA-DR (non-statistical) when compared to responders (Figure S4), similar to specimens collected before the treatment.

Other immune signatures of cancer immune status, namely PD-1, PD-L1, CTLA4, T cell immunoglobulin and mucin domain 3 (Tim3), IDO and IL-10, that potentially could be used as biomarkers of NACT response were analyzed in the pre-treatment biopsies. Although differences could be observed between biopsies of NACT responders and non-responders, none of them was statistically significant, as was HLA-DR in CTLs. Moreover, we did not observe a segregation of these groups of patients according to the level of expression in any of these molecules in the tumor environment (Figure 2D). This emphasizes that HLA-DR^hi^ CTLs (with HLA-DR above the threshold value) could potentially be implemented in the clinic to distinguish NACT responders from non-responders.

### HLA-DR-expressing CTLs are located preferably in intraepithelial tumor structures than in the surrounding normal tissue

Besides the frequency and the type of immune cells in the tumor tissue, their location could also be important to predict patients' clinical outcome (21). For instance, it was found that disease-free survival time is statistically shorter in colorectal carcinoma patients without TILs in the center of the primary tumor mass (22).

**Figure 3:**
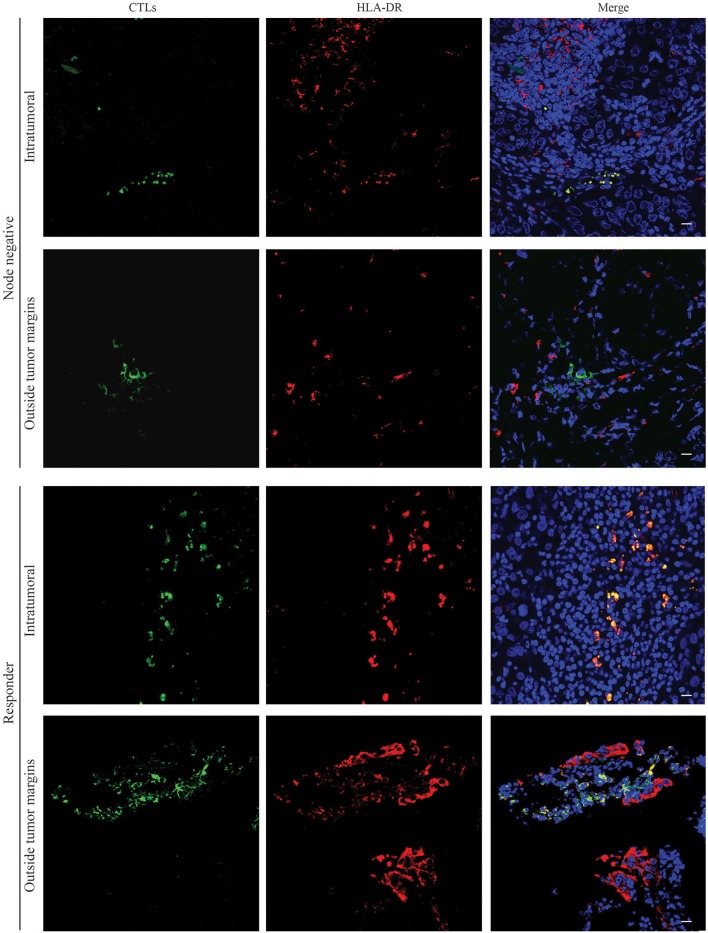
HLA-DR-expressing CTLs are localized in intraepithelial tumor structures of patients without axillary lymph node metastasis and in NACT responders. Representative images of immunofluorescence experiments (*n* = 6) performed in slices of paraffin tissue of surgical BC samples of patients without axillary lymph node metastasis (Node negative) and biopsies of NACT responders (Responders) for CTLs (green, **Left panel**) and HLA-DR (red, **Middle panel**). Nuclei are stained in blue with DAPI and the three staining were merged (**Right panel**). Images from the tumor structures and from the tumor-surrounding tissue were acquired. Scale bars: 20 μm.

Therefore, we have assessed, by immunofluorescence, selected formalin-fixed paraffin embedded (FFPE) tissue samples matched with the previously analyzed by flow cytometry, and evaluated where the HLA-DR+ CTLs are mainly located. We observed that either in surgical samples from patients without axillary lymph node metastasis and in biopsies of patients with good response to NACT (Figure 3), HLA-DR-expressing CTLs were mainly present in intraepithelial tumor structures. Representative images in Figure 3 show a co-localization of anti-CD8 and anti-HLA-DR within the tumor, while no co-localization between these two markers was observed in the tumor surrounding normal tissue. Indeed, outside the tumor margins, CTLs were found, but no co-localization with HLA-DR occurred, as HLA-DR staining was more spread throughout the tissue. These results indicate that HLA-DR+ CTLs are located in intraepithelial tumor structures rather than in the surrounding normal tissue. This may suggest that the presence of HLA-DR+ CTLs in a high proximity with tumor antigens and tumor-released soluble factors, could lead to a more efficient anti-tumor activity.

### Circulating CTLs maintain HLA-DR of tumor infiltrating CTLs in BC patients

**Figure 4:**
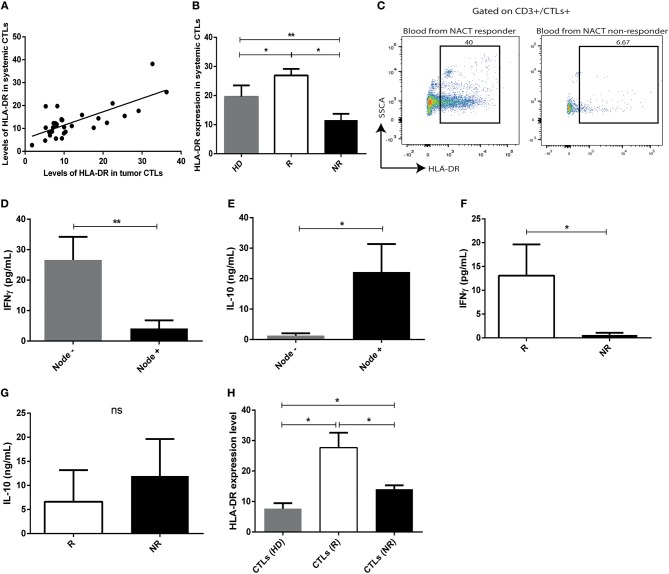
The profile of HLA-DR expression and cytokine production of intratumor CTLs is maintained in systemic CTLs. **(A)** Correlation between expression of HLA-DR in systemic CTLs and tumor infiltrating CTLs (Spearman *r* = 0.58, *p* < 0.001). **(B)** HLA-DR expression in systemic CTLs in healthy donors (HD, gray bar, *n* = 13, mean ± *SD*), NACT responders (R, white bar, *n* = 4, mean ± *SD*) and non-responders (NR, black bar, *n* = 5, mean ± *SD*), assessed by flow cytometry and representing the median fluorescent intensity of positive population normalized relatively to the negative population. **(C)** Representative flow cytometry analysis of HLA-DR in systemic T cells from blood collected at the time of biopsy of responders and non-responders. IFN-γ **(D)** and IL-10 **(E)** levels assessed by ELISA in plasma of patients without axillary lymph node metastasis (gray bar, *n* = 9, mean ± SEM) and with axillary lymph node metastasis (black bar, *n* = 12, mean ± SEM). IFN-γ **(F)** and IL-10 **(G)** levels assessed by ELISA in plasma from responders (R, white bar, *n* = 4, mean ± SEM) and non-responders (NR, black bar, *n* = 6, mean ± SEM). **(H)** Expression of HLA-DR, assessed by flow cytometry, and representing the median fluorescent intensity of positive population normalized relatively to the negative population, in CTLs and Tregs of *ex vivo* stimulated PBMCs isolated from healthy donors (HD, gray bar, *n* = 5, mean ± SEM), from responders (R, white bar, *n* = 4, mean ± SEM) and from non-responders (NR, black bar, *n* = 5, mean ± SEM). *p < 0.05, **p < 0.01, ns, non-statistical.

To verify an association between the tumor immune microenvironment and peripheral blood, blood samples were also collected from 31 patients, prior to treatment implementation, and immunophenotyped by flow cytometry. Intriguingly, we observed that the expression of HLA-DR in tumor infiltrating CTLs correlated with HLA-DR expression in circulating CTLs (*r* = 0.58, *p* = 0.001) (Figure 4A). Moreover, circulating CTLs have higher levels of HLA-DR in NACT responders comparing with non-responders and healthy donors (Figure 4B, *p* < 0.05), therefore allowing the distinction between responders and non-responders (Figures 4B,C). Curiously, non-responders have even lower levels of HLA-DR in circulating CTLs than healthy donors (Figure 4B, *p* < 0.01).

IFN-γ and IL-10 levels in pre-treatment patients' plasma, assessed by ELISA, revealed that patients without axillary lymph node invasion, as well as NACT responders, had higher levels of IFN-γ (*p* = 0.004, *p* = 0.01, respectively, Figures 4D,F) and lower levels of circulating IL-10 (*p* = 0.02, non-statistical, respectively, Figures 4E,G), which is in agreement with the presence of more activated or less activated circulating CTLs, correspondingly.

Peripheral blood mononuclear cells (PBMCs) were isolated from the blood of NACT responders and non-responders, at the time of biopsy, and cultured under a canonical stimulus, to clarify if they could recapitulate the characteristics of the tumor infiltrating immune cells *ex vivo*, when exposed exactly to the same conditions. PBMCs isolated from healthy donors were used as control. Interestingly, the expression of HLA-DR in CTLs was higher in stimulated PBMCs of NACT responders regarding stimulated PBMCs of non-responders (*p* = 0.02, Figure 4H). This observation further supports that the tumor immune features are maintained systemically and blood could be used to specifically assess HLA-DR+ CTLs, which is a major advantage for its potential use as a biomarker of response to NACT.

### HLA-DR-expressing CTLs have increased expression levels of effector immune response-related molecules

**Figure 5:**
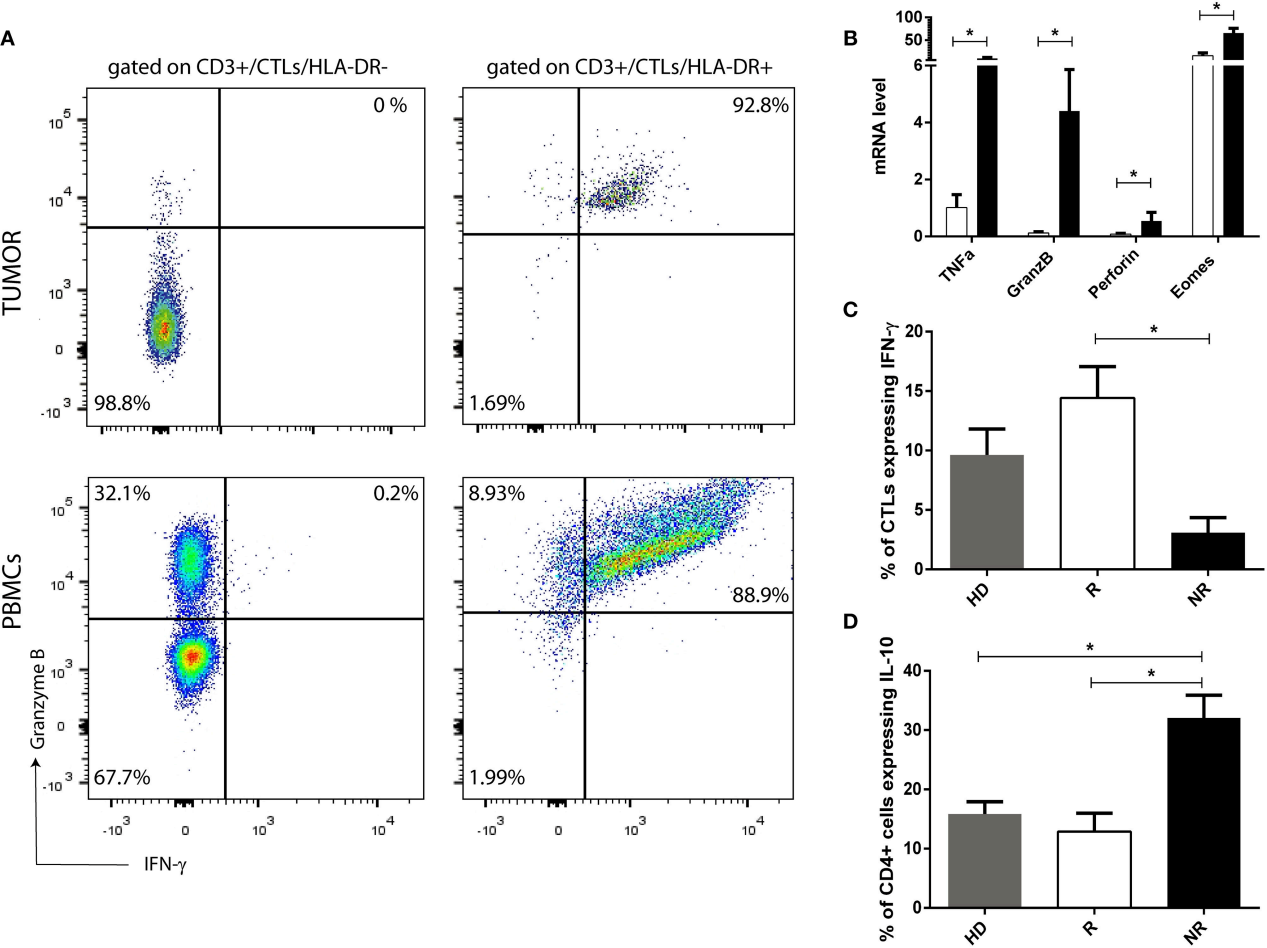
HLA-DR in CTLs reflects their activation and functional status. **(A)** Representation of a flow cytometry analysis of Granzyme B and IFN-γ expression in HLA-DR+ CTLs and in HLA-DR negative CTLs from pre-treatment tumor samples and from PBMCs isolated from patients. **(B)** mRNA level of *TNF*α, *Granzyme B* (*GranzB*), *Perforin* and *Eomes* in HLA-DR+ CTLs (black bars) and HLA-DR negative CTLs (white bars). mRNA level is expressed as the permillage (‰) of the expression of the endogenous positive control (*n* = 4). **(C)** Percentage of CTLs expressing IFN-γ and **(D)** percentage of CD4+ T cells expressing IL-10, assessed by flow cytometry, and representing the median fluorescent intensity of positive population normalized relatively to the negative population, in stimulated PBMCs isolated from healthy donors (HD, gray bar, *n* = 5, mean ± SEM), from NACT responders (R, white bar, *n* = 4, mean ± SEM) and from non-responders (NR, black bar, *n* = 5, mean ± SEM). *p < 0.05.

To corroborate that HLA-DR+ CTLs are activated lymphocytes with an anti-tumor activity profile, we assessed in pre-treatment samples, infiltrated HLA-DR+ CTLs and HLA-DR negative CTLs, the expression, by flow cytometry, of other effector immune response-related molecules, such as IFN-γ and Granzyme B, which are the most important players of activated CTLs. Interestingly, only HLA-DR+ CTLs expressed high levels of IFN-γ and Granzyme B (Figure 5A), substantiating their immune competent profile. Furthermore, we have analyzed the expression level of these molecules also in HLA-DR+ CTLs and HLA-DR negative CTLs from patients PBMCs (isolated from pre-treatment blood). Similar to tumor, circulating HLA-DR+ CTLs express more IFN-γ and Granzyme B than HLA-DR negative CTLs (Figure 5A). Even if HLA-DR negative CTLs from PBMCs had a higher level of Granzyme B expression when compared to HLA-DR negative CTLs from tumor, the percentage of HLA-DR+ CTLs from PBMCs that express Granzyme B, and the corresponding median fluorescent intensity, is significantly higher than in HLA-DR negative CTLs from PBMCs (*p* < 0.01, data not shown).

Additionally, to further understand differences between HLA-DR+ CTLs and HLA-DR negative CTLs, CTLs with and without HLA-DR expression isolated from the blood were studied by qRT-PCR. In this case, the expression of *Granzyme B, Perforin, TNF*α and *Eomes* were assessed. HLA-DR+ CTLs expressed higher levels of the cytolytic proteins *Granzyme B* (*p* = 0.02) and *Perforin* (*p* = 0.03); *Eomes* (*p* = 0.03), involved in differentiation of effector CTLs and the inflammatory cytokine *TNF*α (*p* = 0.03), which also has anti-tumor properties, in comparison with HLA-DR negative CTLs (Figure 5B).

Also, *ex vivo* stimulated PBMCs of responders (shown to express more HLA-DR in CTLs than PBMCs of non-responders—Figure 4H) have increased levels of IFN-γ (*p* = 0.01) in CTLs and lower levels of IL-10 (*p* = 0.01), produced by Th cells and/or Tregs respectively (Figures 5C,D), than stimulated PBMCs of non-responders.

Altogether, these results suggest that CTLs with expression of HLA-DR are active and effector lymphocytes that might have a protective anti-tumor effect, which is in accordance to their higher prevalence in BC without axillary lymph node metastasis and/or in NACT responders.

### HLA-DR expression level in CTLs negatively correlates with the immunosuppressive and pro-tumor features of the tumor milieu

**Figure 6:**
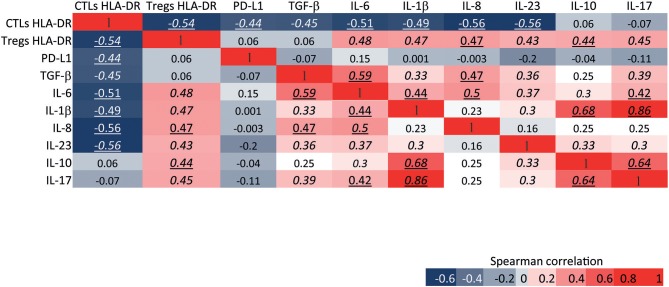
HLA-DR expression level in CTLs and Tregs is differentially correlated with the immunosuppressive and pro-tumor features of the tumor environment. Heat map of Spearman correlations between the expression level of HLA-DR in CTLs and the expression level of HLA-DR in Tregs; and between the expression level of HLA-DR in both of these cells and the expression level of pro-tumor or immunosuppressive molecules from the tumor environment, namely PD-L1, TGF-b, IL-6, IL-1b, IL-8, IL-23/IL-12, IL-10, and IL-17. The expression level of each molecule was assessed by flow cytometry and represents the median fluorescent intensity of positive population normalized relatively to the negative population. The heat map is represented as a gradient from blue (negative correlations) to red (positive correlations). Numbers in italic represent statistical significance of *p* < 0.01, numbers underlined represent *p* < 0.001 and numbers in italic and underlined represent *p* < 0.0001.

An anti-tumor or a pro-tumor immune response is elicited not only by the immune cells within the tumor and their bioeffector molecules, but also by tumor antigens and the expression of cytokines, chemokines and other immune mediators released by the cancer tissue. Thus, the stimulation of anti-tumor CTLs' activity should, at least in part, rely on the molecules present in the tumor milieu. Interestingly, we have observed, by flow cytometry analysis of pre-treatment BC samples, that CTLs expressing HLA-DR were inversely correlated with immunosuppressive activated Tregs (also expressing HLA-DR, Figures 1E, 6). Moreover, they were also negatively correlated with the expression of molecules from the non-immune compartment that may co-opt the expression of typically innate immune system-associated molecules to squelch the anti-tumor immune program and/or enhance growth and survival of cancer cells (Figure 6). Namely, HLA-DR expression level in CTLs was negatively correlated with the expression level of TGF-β (*r* = −0.45, *p* < 0.01), which increases the metastatic profile; PD-L1 (*r* = −0.44, *p* < 0.0001), that inhibits infiltration and/or activation of CTLs (23, 24); IL-6 (*r* = −0.51, *p* < 0.001), IL-8 (*r* = −0.56, *p* < 0.001) and IL-1β (*r* = −0.49, *p* < 0.001) that are inflammatory cytokines that may act as growth factors to sustain cancer cell proliferation and invasion (25–27). It was also negatively correlated with IL-23/IL-12 (*r* = −0.56, *p* < 0.0001) that is pro-inflammatory but can also impair CTLs' activity through the activation of Tregs (28) (Figure 6). On the other hand, the expression level of HLA-DR in Tregs was positively correlated with the expression of these pro-tumor molecules - IL-1β (*r* = 0.47, *p* < 0.01), IL-23/IL-12 (*r* = 0.43, *p* < 0.01), IL-6 (*r* = 0.48, *p* < 0.01) and IL-8 (*r* = 0.47, *p* < 0.001) – and, additionally, IL-10 (*r* = 0.44, *p* < 0.0001) and IL-17 (*r* = 0.45, *p* < 0.01), which could also undermine the activation of CTLs and lead to tumor progression (29, 30) (Figure 6).

When the same analysis was performed with CTLs or Tregs without accounting for HLA-DR expression, there was no significant correlation between these cells and these molecules of the tumor milieu (results not shown).

To better understand the contribution of cytokines or other soluble factors present in the tumor milieu in the activation of CTLs, we used a co-culture assay of HS 578T breast cancer cell line with peripheral blood mononuclear cells (PBMCs) isolated from patients. The assay was performed in several conditions: PBMCs with HS 578T cell line, PBMCs with the supernatant of the cell line alone, PBMCs plus a canonical stimulus, which served as a positive control, or just PBMCs. Curiously, the supernatant of the cell line alone increased the expression of HLA-DR in CTLs (Figure S5).

Altogether, these data are in agreement with the supposition that CTLs (and Tregs) bearing HLA-DR reflect the overall immune status of the BC milieu.

## Discussion

Chemotherapy failure is the main reason for disease progression, recurrence and cancer-related death. In the case of breast cancer (BC), less than 50% of the patients have a pathological complete response to neoadjuvant chemotherapy (NACT) (3, 4). Although some efforts have been made in the past years, such as the introduction of tumor infiltrating lymphocytes (TILs) as possible biomarkers of response, especially in TNBC and HER2+ BC (7), there is still no validated biomarker being routinely used in the clinic.

The belief that NACT outcome may depend on the presence of TILs is related to the fact that chemotherapy leads to the release of immunogenic signals, promoting immunogenic cell death, which can potentially boost an anti-tumor immune response (31). However, TILs are functionally heterogenic. For instance, cytotoxic T lymphocytes (CTLs) have been strongly associated with patient survival and response to therapy (7); regulatory T cells (Tregs) have been associated with both good and bad prognosis (6, 9); T helper 1 (Th1) cells have been related with favorable clinical outcomes (32); whereas Th2 cells have been reported to be associated with dampening of the anti-tumor response (33). Therefore, it is controversial that simply the degree of lymphocytic infiltration, assessed by immunohistochemistry in sections of paraffin embedded tissue, has a predictive value of BC patients' response to NACT. Even infiltrating CTLs, *per se*, lack the robustness that a predictive biomarker should have, because their function can be hampered by tumor cells, through several mechanisms. For instance, by expressing inhibitory molecules like PD-L1 or by secreting immunosuppressive molecules such as IL-10, TGF-β and IDO.

Thus, it is still crucial to establish a more accurate BC “immunological status” that reflects the overall strength of individual patients' anti-tumor immune response that will ultimately influence NACT efficiency.

In this study, we went beyond immunohistochemistry evaluation of TILs in BC and analyzed, by flow cytometry, T lymphocyte immune signatures, that could show their activated/exhausted or anergic state and molecules present in the tumor environment that are representative of the global tumor immune status.

We found that CTLs expressing high levels of HLA-DR, mainly located in intraepithelial tumor structures, are characteristic of BC without axillary lymph node metastasis and are strongly associated with good response to NACT treatment, contrasting with CTLs with low or null expression of this molecule. HLA-DR is an antigen presenting molecule expressed at high levels on professional antigen presenting cells, but its expression on effector T lymphocytes upon their activation has also been intensively described in some diseases, such as auto-immune diseases and viral infections (14, 15).

In the context of HIV patients, it has been described that these HLA-DR+ CTLs exhibit a decreased proliferative potential although retaining their cytolytic potential (34, 35). Additionally, in severe aplastic anemia, an elevated number of HLA-DR+ CTLs detected in patients' peripheral blood are responsible for the excessive apoptosis of hematopoietic cells (36). However, a suppressive effect of a subset of HLA-DR+ CTLs was also reported (37). Here we confirmed that HLA-DR+ CTLs express immune signatures of functionally activated, and not suppressive CTLs, such as the production of IFN-γ, Granzyme B, Perforin, TNFα, and Eomes. Thus, the infiltrating CTLs bearing HLA-DR found in the analyzed samples should have an active participation in the anti-tumor response, in conformity with the fact that they were more elevated in patients without axillary lymph node metastasis and with a better capacity to respond to NACT.

Tregs are recognized blockers of CTLs' function and it is known that HLA-DR also identifies functionally mature Tregs (13). Thus, not surprisingly, HLA-DR expression level in Tregs negatively correlates with its expression level in CTLs and is associated with BC with axillary lymph node metastasis and more abundant in biopsies of NACT non-responders.

In line with these former results, we also presented data that negatively correlate the level of HLA-DR in CTLs with the level of IL-6, IL-1β, IL-8, and IL-23/IL-12, which are inflammatory cytokines that, at certain level in the tumor microenvironment, could help the anti-tumor immune response (38), but whose upregulation might also favor tumor progression and metastasis (25–28). HLA-DR in CTLs was also inversely correlated with the level of PD-L1 and TGF-β, which are, respectively, a well-known inhibitory immune checkpoint and an anti-inflammatory cytokine, which coordinately work to dampen CTLs' activity, directly or through the activation of Tregs (23, 24). Likewise, HLA-DR expression in Tregs positively correlated with all the aforementioned molecules from the tumor milieu and, additionally, with IL-10 and IL-17, which also have immunosuppressive functions (29, 30, 39).

*In vitro* experiments, with the supernatant of BC cell lines, highlighted the fact that soluble factors released by tumor cells are important to increase HLA-DR in CTLs, suggesting that these tumor microenvironment molecules can modulate the immune response and influence the activation state of CTLs, without the requirement of contact with tumor cells.

In the other way around, we may say that the HLA-DR expression in CTLs is a reflection of the general tumor immune status. Indeed, an immunosuppressed environment will be unable to stimulate an appropriate immune response and should not give rise to HLA-DR^hi^ CTLs. As cell-to-cell contact and tumor-specific antigen presentation by antigen presenting molecules are required to elicit a specific cytotoxic T cell response against the tumor cells, we may infer that, *in vivo*, the antigens presented by tumor cells, alongside with the soluble factors, contribute to CTLs' effector function.

The increase of HLA-DR at CTLs' surface, upon stimulation, could also be required to boost the anti-tumor immune response. Indeed, HLA-DR+ CTLs were found to have the machinery needed for antigen processing and loading on HLA-DR molecules and, additionally, could express CD86 and CD80, which are the co-stimulatory molecules of antigen presenting cells that are necessary for the proper T cell effector function (40). Moreover, it was described that T cell-T cell synapsis occur to allow T cells to secrete IFN-γ toward each other, compelling the differentiation of more protective T cells (41). These T cell-T cell interactions and mutual antigen presentation can be essential for mounting a suitable anti-tumor response.

Notably, we observed that HLA-DR expression on tumor infiltrating CTLs correlates to its expression in circulating CTLs and, additionally, the analysis of HLA-DR level on CTLs isolated from the patients' blood could also be associated with BC axillary lymph node invasion status and assist in the prediction of patients' response to NACT. *Ex vivo* assays indeed corroborate that systemic CTLs maintain the profile encountered in the tumor mass, as under similar stimulation, CTLs from NACT responders increase even more their HLA-DR level and produce more IFN-γ than CTLs from NACT non-responders. These results imply that tumor immune status, reflected by the HLA-DR level in CTLs, could also be easily assessed in blood.

HLA-DR molecule, by itself, assessed in tumor cells, has been reported to serve as a favorable prognostic marker for other types of cancers, such as colorectal carcinoma (42) or glioma (43). It was even shown that HLA-DR+ melanoma cells predict the response to anti-PD-1/PD-L1 immunotherapy (44). However, to our knowledge, the expression of HLA-DR in CTLs, besides its extensive application in the study of viral infections and chronic inflammatory diseases, was never recommended as a biomarker in cancer. Our data suggest that HLA-DR+ CTLs, assessed by flow cytometry, in BC patients prior to NACT, is a sensitive and specific potential predictive factor for NACT response. Moreover, this trait can be easily assessed in blood, repeatedly if necessary. Further studies should be conducted in an independent population, in order to validate the predictive performance of HLA-DR+ CTLs in assisting the selection of patients that will truly benefit from NACT, or promptly directing them to alternative therapeutic strategies, such as the combination of immunotherapies with standard chemotherapy. Additionally, this marker also have the potential to be used in clinical trial design to randomize good/bad responders to evaluate novel treatments.

## Author contributions

DS conducted all the experiments, analyzed and interpreted the data, performed the statistical analysis, assembled all the figures and wrote the manuscript. AJ contributed to scientific discussion and revised the manuscript. PB helped in the obtainment of patients' samples and clinical data, contributed to scientific discussion and revised the manuscript. SB supervised the study, helped in the obtainment of patients' samples and clinical data, contributed to data interpretation, scientific discussion and revised the manuscript. MC conceived and designed the experiments, supervised the study, analyzed and interpreted the data and wrote the manuscript. All the authors approved the final manuscript.

### Conflict of interest statement

The authors declare that the research was conducted in the absence of any commercial or financial relationships that could be construed as a potential conflict of interest.
