# Supplementary material for: HLA-DR in Cytotoxic T Lymphocytes Predicts Breast Cancer Patients' Response to Neoadjuvant Chemotherapy
Source: Front Immunol. 2018 Nov 13;9:2605. doi: 10.3389/fimmu.2018.02605 (PMC6282034; doi:10.3389/fimmu.2018.02605)
Supplement: Supplementary file 1 [file Data_Sheet_1.docx]

**Supplementary material**

**Fig. S1**


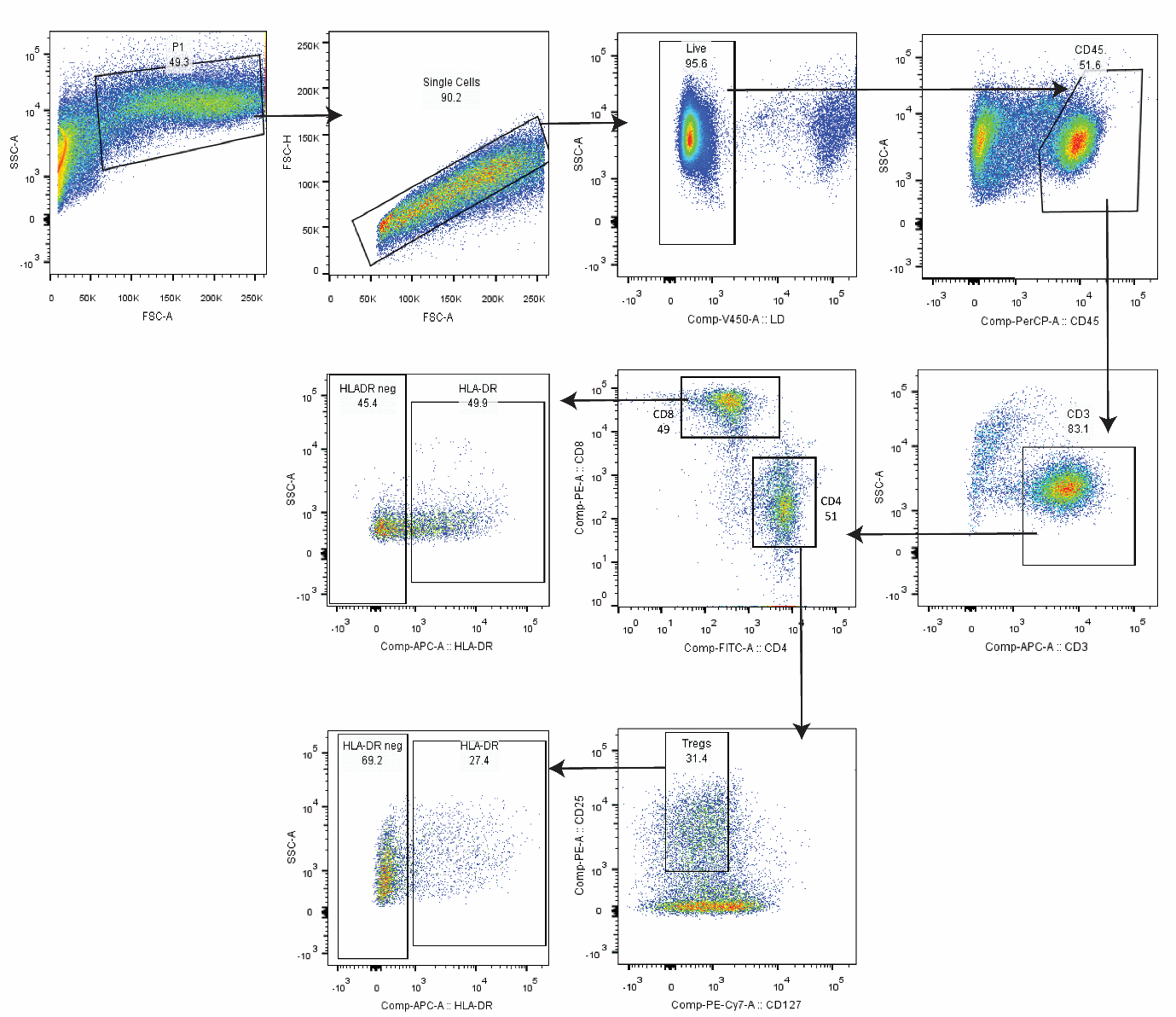


**Supplementary figure S1** – Representative dot plots of the gating strategy used in the flow cytometry analysis of breast cancer samples. Positive and negative populations were always selected taken into consideration the negative (unstained) sample.

**Fig. S2**


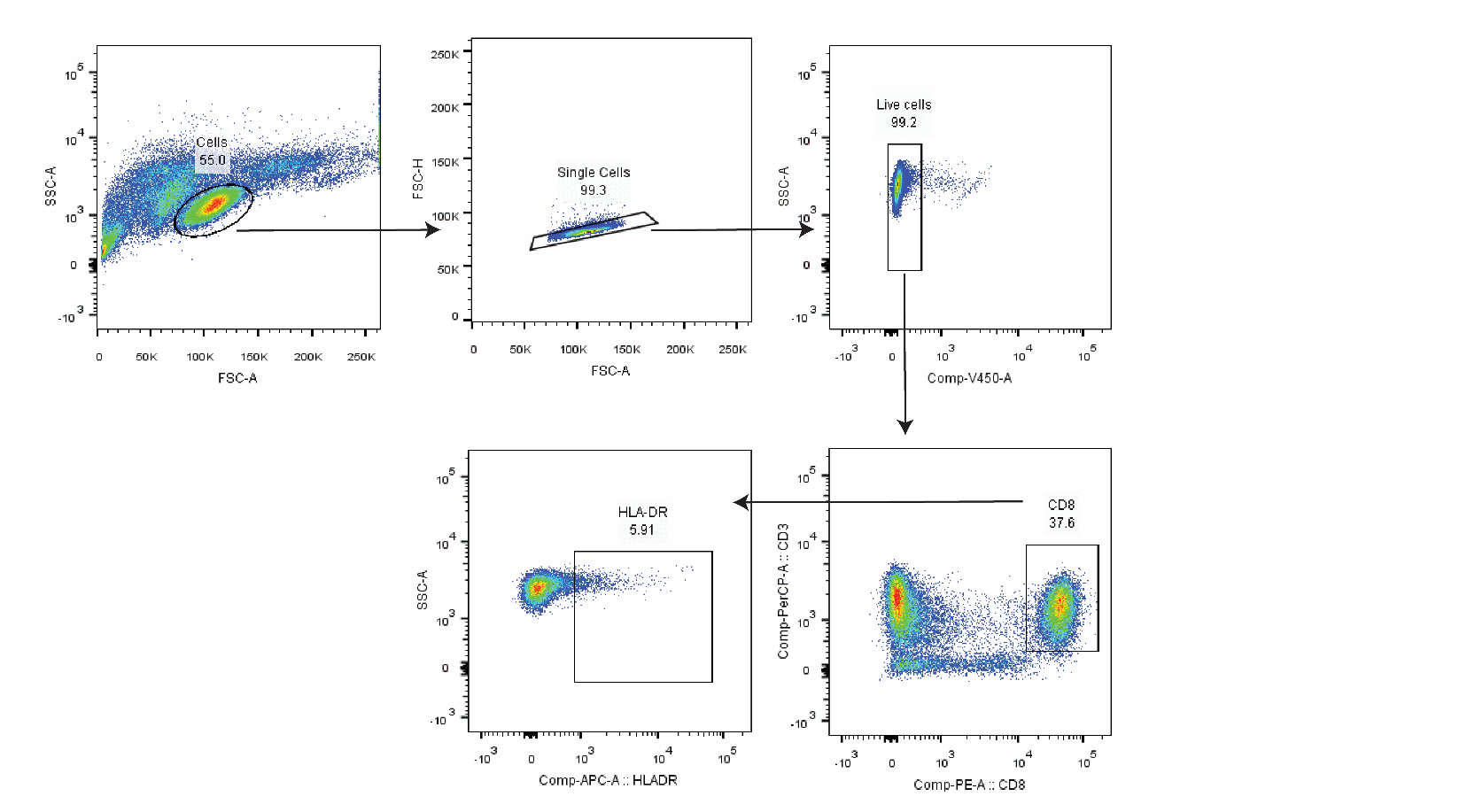


**Supplementary figure S2 -** Representative dot plots of the gating strategy used for fluorescence activated cell sorting of CTLs positive and negative for HLA-DR. These cells were then used for qRT-PCR analysis.

**Fig. S3**

**
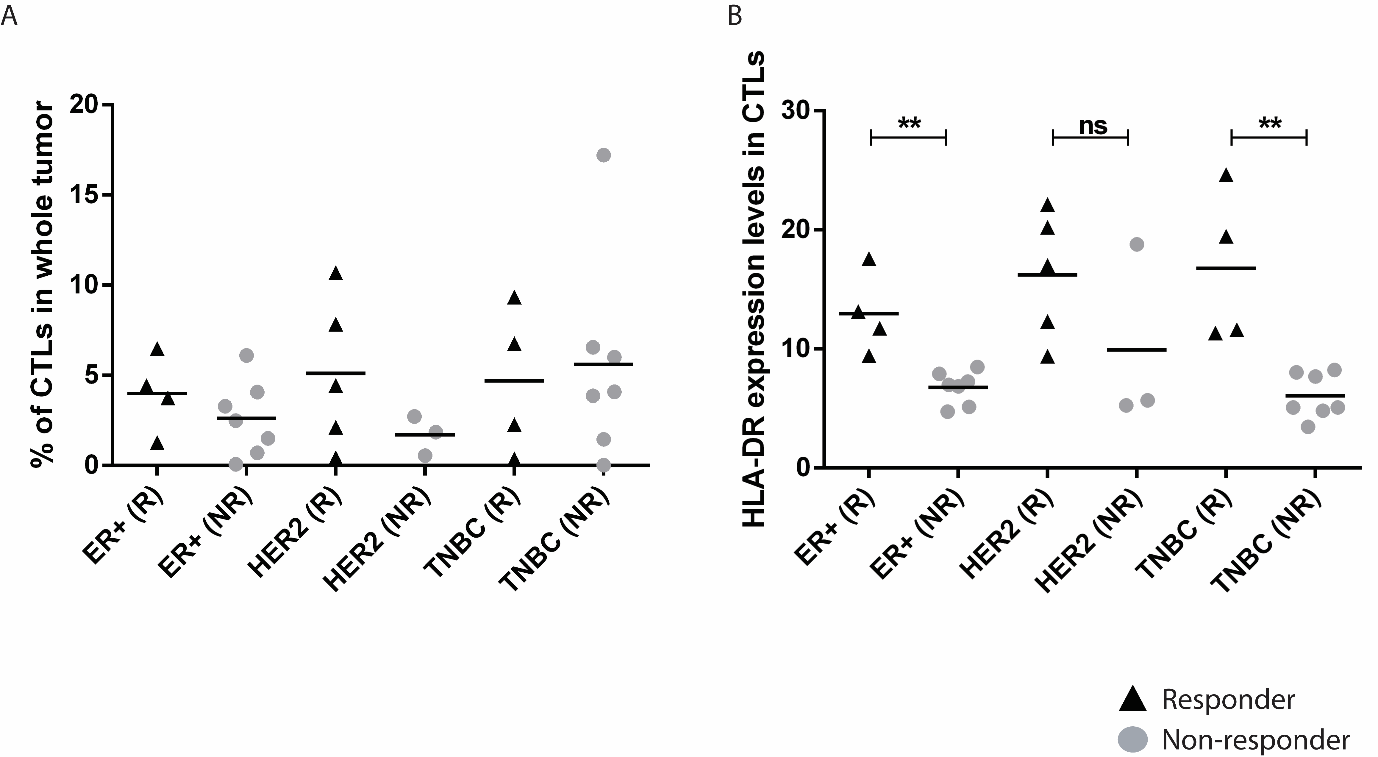
**

**Supplementary figure S3 – HLA-DR levels in CTLs can predict response to NACT, independently of breast cancer subtype. (A)** Percentage of CTLs in pre-treatment biopsies of NACT-responders (R, black triangles) and non-responders (NR, grey dots) divided in the three breast cancer subtypes – Estrogen receptor positive (ER+), HER2 amplified (HER2) and triple negative breast cancer (TNBC). **(B)** HLA-DR expression levels in CTLs in responders and non-responders for the three subtypes of breast cancer. The expression level of HLA-DR was assessed by flow cytometry and represents the median fluorescent intensity of positive population normalized relatively to the negative population. **p<0.01, ns – non statistical.

**Fig. S4**


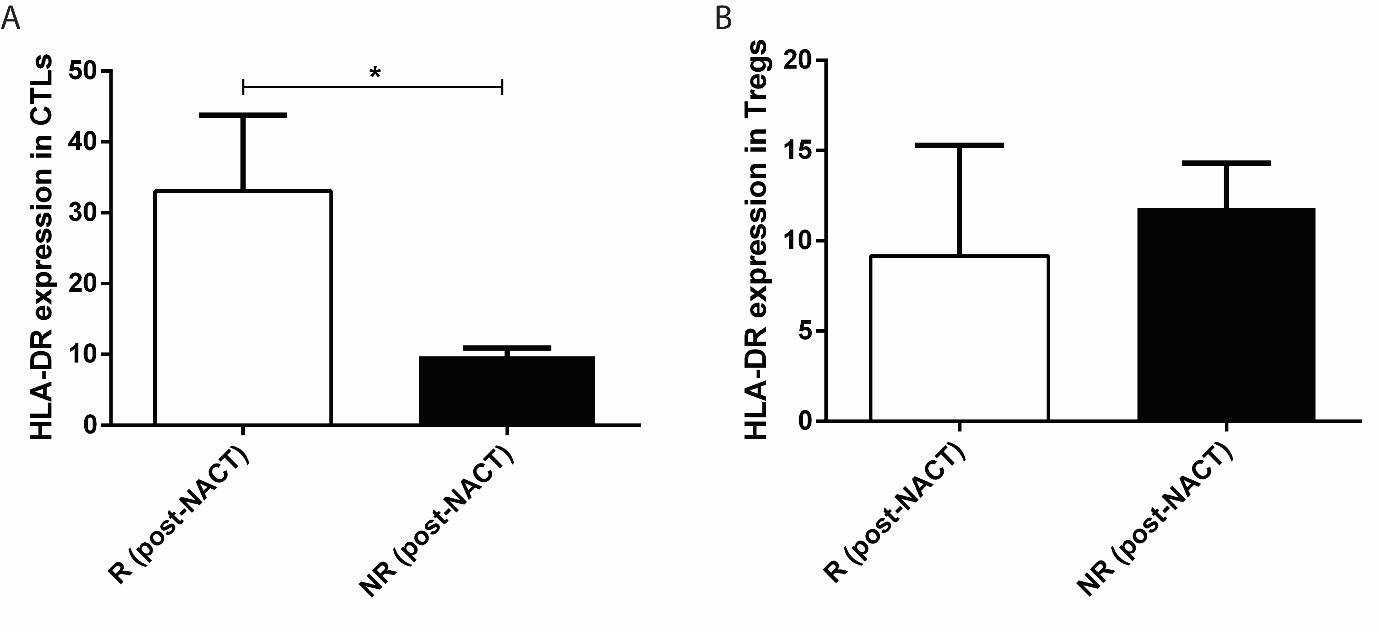


**Supplementary figure S4 – Profile of HLA-DR expression in CTLs and Tregs after neoadjuvant chemotherapy (NACT).** Level of HLA-DR in CTLs **(A)** and Tregs **(B)**, assessed by flow cytometry, in responders (R, white bars, n=3, mean ± SEM) and non-responders (NR, black bars, n=4, mean ± SEM), expressed as the median fluorescent intensity of positive population normalized relatively to the negative population. *p<0.05.

**Fig. S5**


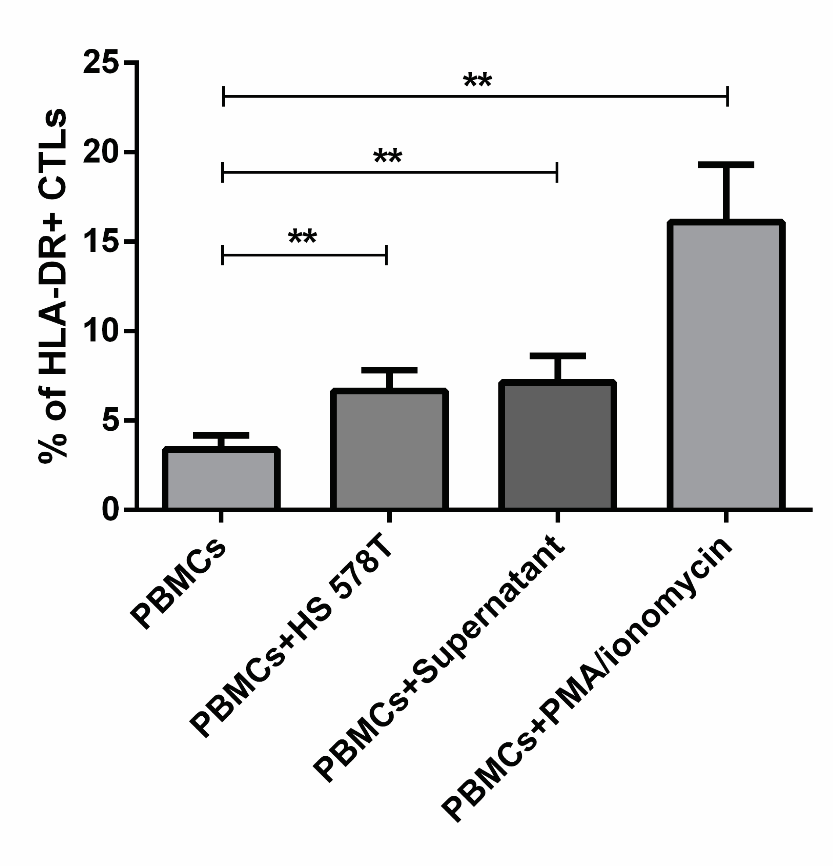


**Supplementary figure S5 – HLA-DR expression in CTLs in a co-culture of PBMCs with a breast cancer cell line.** HLA-DR+ CTLs, assessed by flow cytometry, in the following conditions: PBMCs in mono-culture, co-culture of PBMCs with HS 578T cell line, PBMCs with the cell line supernatant and PBMCs with the canonical stimulus (PMA/ionomycin). Data are expressed as the percentage of HLA-DR+ CTLs in these conditions (**p<0.01, n=6).
